# Hamstring Muscle Injuries: MRI and Ultrasound for Diagnosis and Prognosis

**DOI:** 10.5334/jbsr.2617

**Published:** 2022-03-25

**Authors:** Carles Pedret

**Affiliations:** 1 Clinica Diagonal, ES

**Keywords:** Hamstring, muscle injuries, MRI, ultrasound, return to play

## Abstract

Hamstring injuries (HSI) are the most frequent muscle injuries in active individuals and professional athletes. Early and accurate diagnosis is key for planning a correct and individualised return to play (RTP). For that diagnosis imaging tests such as Magnetic Resonance Imaging (MRI) and ultrasound (US) are the most useful tests in the initial stages.

## Introduction

Hamstring injuries (HSI) are frequent muscle injuries in active individuals and professional athletes. According to a recent study in English football [1], thigh muscle strains were the most commonly injured region, and hamstring was the most frequently injured muscle group, accounting for 39.5% of all muscle strains and 16.3% of all injuries.

The particular anatomy of this muscle group with large intramuscular connective expansions, as well as the fact that none of these muscles have a uniform architecture, gives the hamstrings a certain structural complexity that makes it difficult to approach in terms of imaging diagnosis and prognosis [2,3].

An acute HSI is immediately perceived by the athlete, and he/she reports localised pain and tenderness and is not able to continue with his/her normal activity. In the first 24–48 hours the main objective should be to have a fast, concrete, and accurate diagnosis in order to plan a return-to-play (RTP) process as individualized as possible to the athlete and to the specific injury [4,5].

## HSI Types and Imaging Methods. Defining the Injury Type

**Figure 1:**
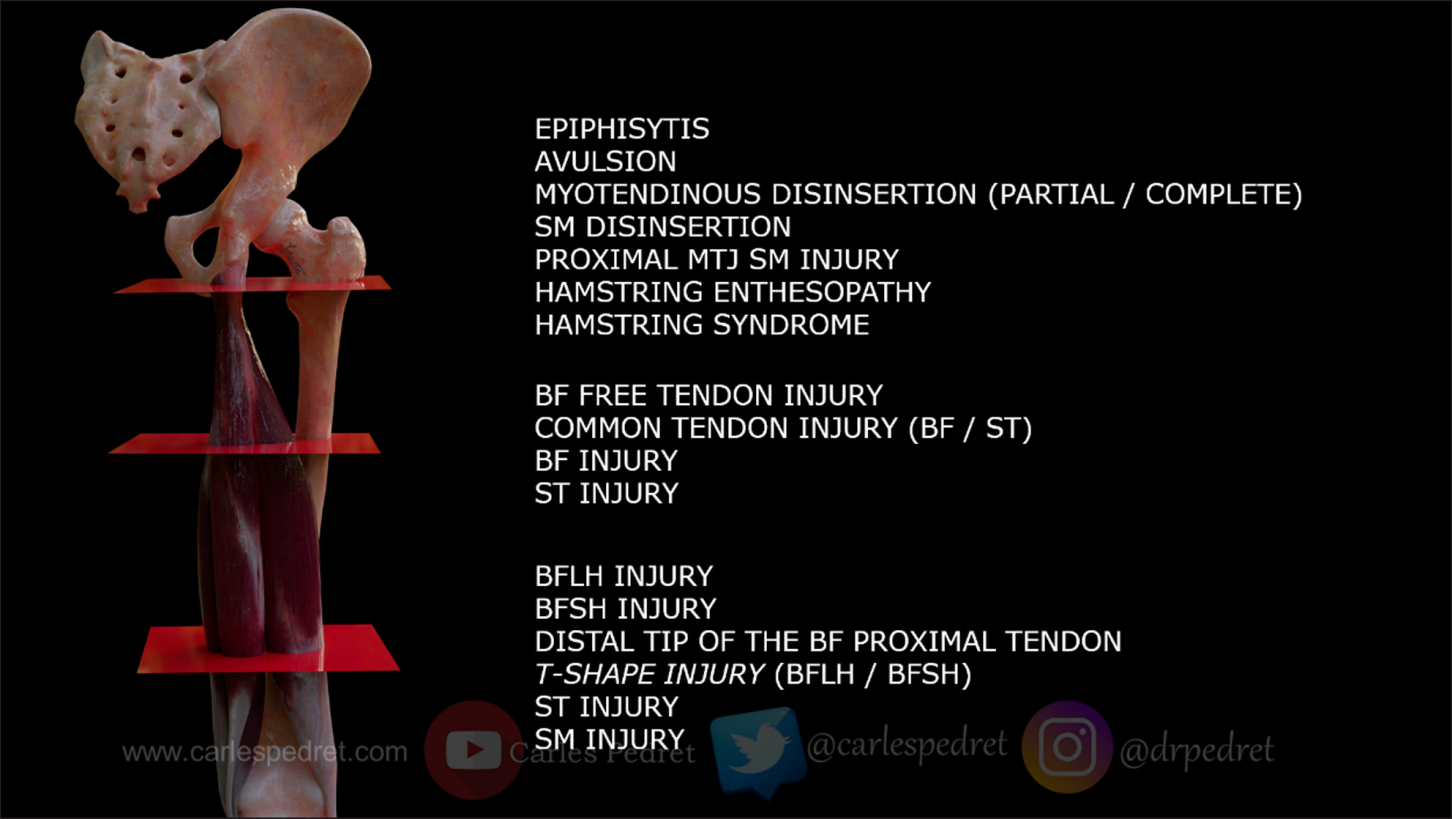
All HSI types distributed by region. Origin region, proximal region and distal region. SM = semimembranosus; BF = biceps femoris; ST = Semitendinosus; BFLH = biceps femoris long head; BFSH = biceps femoris short head.

There is a wide variety of muscular injuries in the hamstring group and they can be divided into regions (origin, proximal, and distal) (***Figure 1***), but the most important aspect to take into account is the exact location and the amount of connective tissue affected [6,7,8] because this is the most important prognosis factor to plan a correct treatment.

For this, an early and accurate diagnosis is essential, and the most appropriate tools available in the first 72 hours are magnetic resonance imaging (MRI) and ultrasound (US), each with its limitations and strengths, which are necessary to know and to understand. Needless to say, clinical examination, knowledge of the mechanism of injury, and exploratory tests are also vitally important [9].

In the first 24 hours the best imaging method is likely to be MRI, and US can be useful after 72 hours and in the follow up. It is also important to know that US has some limitations when assessing the HSI, especially in the origin injuries (some longitudinal splits of the biceps femoris free tendon and common tendon) and in assessing the severity of reinjuries due to the quality and the echo pattern of the tissue (soft scars).

**Figure 2:**
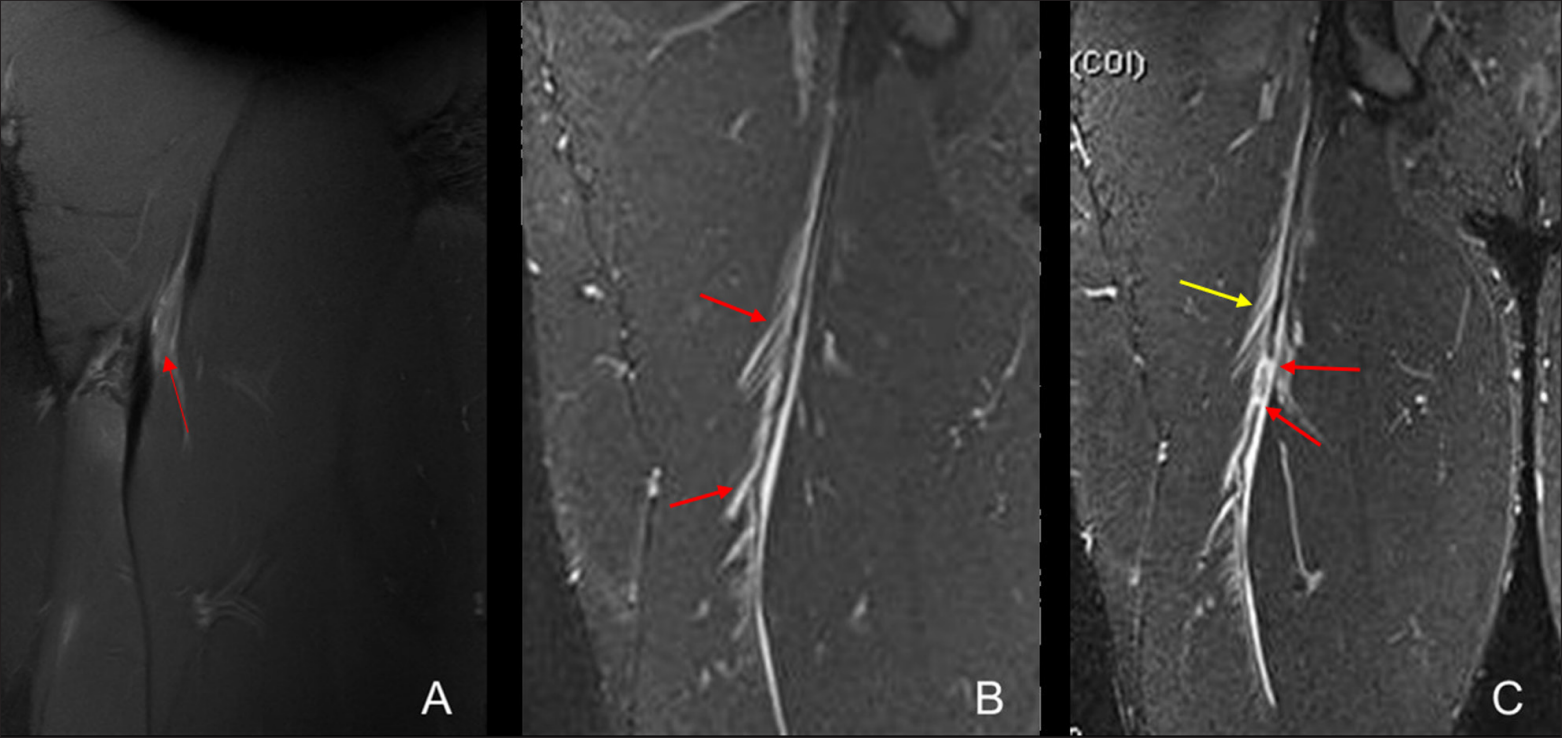
Coronal fat-suppressed proton density-weighted (**a**) and Short-Tau Inversion-Recovery (**b** and **c**) MRI of the right thigh of different patients. Complete tear of the free tendon in healing process (red arrow) (A), Myotendinous junction (MTJ) injury without tear of the BFLH connective tissue (red arrows) (B) and MTJ (yellow arrow) injury with mixed tear (transversal and longitudinal) of the central tendon (red arrows).

The information that MRI provides in the first 24 hours after the injury allows, in most cases, to make a precise prognosis of the RTP time. This is because MRI can define whether the injury is an injury with a great tendon affection, if it has musculotendinous involvement with fascial characteristics, or if it’s “purely” muscular. MRI can even provide information on whether there are combinations of different “anatomical” types of injuries (***Figure 2***).

Each of these injury types has a different prognosis and a different progression of rehabilitation exercises because the type of tissue affected is different.

If there is a need to categorize which are the most important injuries in terms of prognosis, it could be said that those with a worse prognosis would be, in this order: the injury to the BF free tendon and injuries to the common tendon (not only due to the initial severity but also due to the high recurrence rate). After these, a group could be made with injuries that affect the T-shape (between the BFLH and BFSH) and those that affect the distal tip of the proximal BF tendon [10,11,12,13].

**Figure 3:**
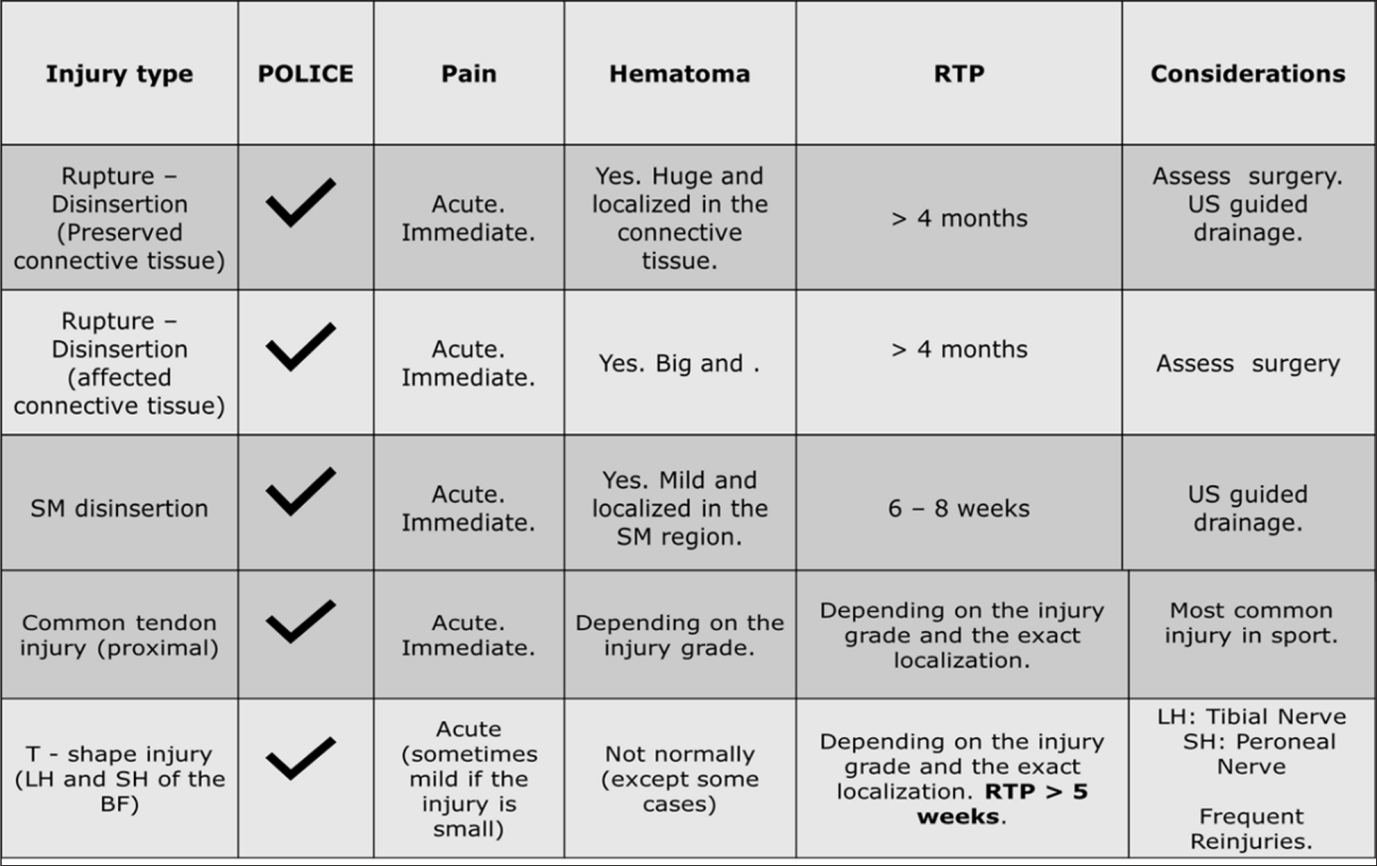
Summary of some of the most common injuries in the Hamstring muscle group and its assessment.

Surgery should always be considered and the RTP is also usually longer than four months with the complete detachments and avulsions in professional and young athletes (***Figure 3***).

## Conclusions

HSI should not be standardized, neither in treatment nor in prognosis. The best prognostic factor and what gives more relevant information is the exact location of the muscle injury and the anatomical tissue structures that are affected inside it.

Myoconnective architecture is of great importance, especially regarding the prognosis of the injury (the more connective tissue affected, the worse the prognosis).

Based on a precise diagnosis, athletes with muscle injury and associated connective tissue lesions should be treated with specifically tailored methods. This diagnosis can be made within the first 72 hours with imaging methods such as MRI and US.

It is essential to have an early diagnosis as accurate, secure, and concrete as possible because we can’t treat what we don’t know. It is also essential to have a unified nomenclature, so that every person in the medical staff (doctors, physiotherapists, physical trainers) can understand exactly what type of injury has been sustained.
